# Additional use of anti-rotation U-blade (RC) decreases lag screw sliding and limb length inequality in the treatment of intertrochanteric fractures

**DOI:** 10.1038/s41598-021-96988-z

**Published:** 2021-08-31

**Authors:** Seungbae Oh, Yong-Sik Kim, Soon-Yong Kwon, Jiyoung Jung, Chiyoung Yoon, Joo-Hyoun Song

**Affiliations:** 1 Department of Orthopaedic Surgery, St. Vincent’s Hospital, The Catholic University of Korea, Suwon, Gyeonggi-do Republic of Korea; 2 Department of Orthopaedic Surgery, Seoul St. Mary’s Hospital, The Catholic University of Korea, Seoul, Republic of Korea; 3 Department of Orthopaedic Surgery, Eunpyeong St. Mary’s Hospital, The Catholic University of Korea, Seoul, Republic of Korea

**Keywords:** Anatomy, Diseases, Health care, Health occupations, Medical research

## Abstract

The purpose of this study is to compare the cut-out rate and sliding distance associated with limb length inequality between operations using a standard non-sliding lag screw versus those using a non-sliding lag screw with U-blade (RC) in the Gamma3 nail. This is a retrospective review of two case series involving different lag screws for the Gamma3 nail. Propensity score matching analysis was used to adjust the confounding factors. A comparative analysis of 304 patients who treated with Gamma3 nail with either a standard non-sliding lag screw or a U-Blade (RC) lag screw was performed. Between 2014 and 2018, 152 patients were treated with U-blade (RC) lag screws, and these patients were matched with those treated with standard lag screws. There was no significant difference in cut-out rate between groups. However, additional use of anti-rotation U-blade (RC) could significantly decrease lag screw sliding, with the group treated with U-Blade (RC) lag screws exhibiting shorter sliding, especially in AO/OTA31 A2 and A3 fractures. Also, in A2 and A3 fractures, the mean lag screw sliding distance was greater than that seen in A1 fractures in both groups. These findings can help trauma surgeons choose the proper implant to reduce leg length inequality.

## Introduction

Hip fractures are the most common type of fractures requiring hospitalization, followed by distal radius fractures and ankle fractures^1^. According to the literature, the incidence of hip fractures differs depending on country and race, though the difference between western and eastern areas is narrowing^2^. Understanding the incidence, management, and outcome of hip fractures is a vital step in improving population health^3^.

Intertrochanteric fracture and femoral neck fracture represent the majority of hip fractures and occur with similar frequency^4^. Perioperative multidisciplinary care is important in regard to osteoporosis assessment and treatment as well as to postoperative functional mobility. Evidence-based management of intertrochanteric fractures includes internal fixation with intramedullary nails or sliding hip screws. Proximal femur intramedullary nails are frequently used for fixation of intertrochanteric fracture. The Gamma3 Nail (Trochanteric Nail 170; Stryker Trauma GmbH, Schoenkirchen, Germany) is a commonly used option.

**Figure 1:**
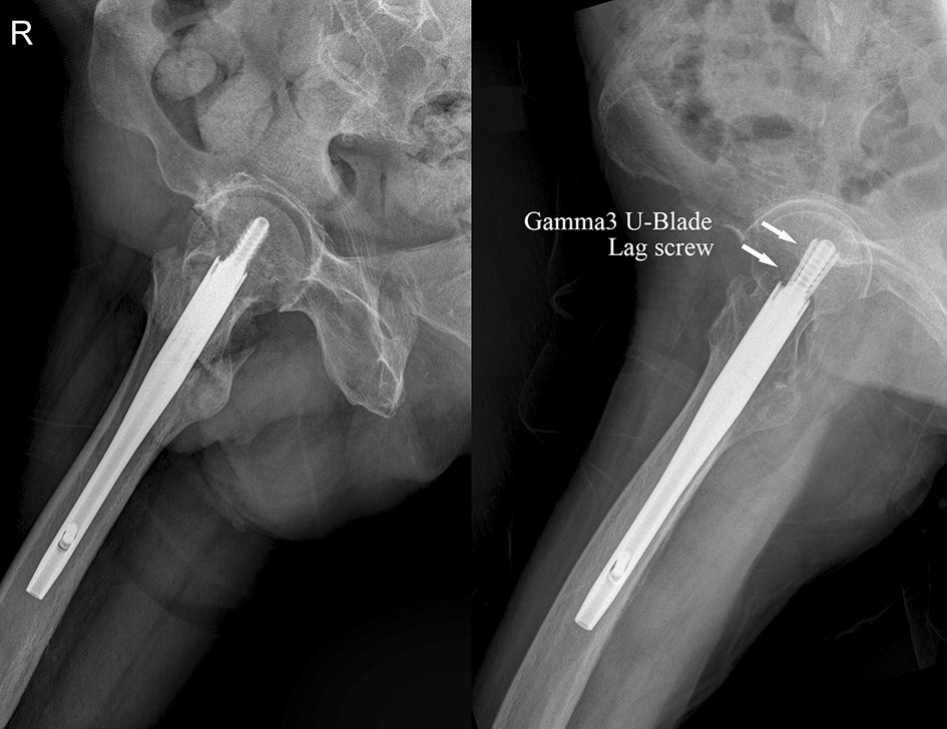
Standard lag screw and U-blade (RC) lag screw.

A model of Gamma3 nail with a U-blade lag screw was developed to provide improved outcomes when treating unstable proximal femur fractures and those at high risk of rotation (Fig. 1). However, evidence suggesting improved outcomes with the use of the U-blade lag screw compared with conventional Gamma3 nails is limited.

A biomechanical study done to evaluate different lag screws showed that a U-blade can widen the surface of the lag screw and increase the volume, eventually blocking migration through an increase in friction force. The use of an RC hip screw significantly increased the resistance of the Gamma3 hip screw by almost 15%^5^. In another biomechanical study, a screw-blade hybrid type lag screw was more effective in minimizing rotation instability of the proximal fragment. Also, varus collapse of the proximal fragment and cranial and axial migration within the femoral head were reduced with screw-blade hybrid type^6^.

However, most clinical studies had a negative view on the usefulness of U-blade. In one study, the U-Blade (RC) lag screw did not reduce the cut-out in treatment of intertrochanteric fractures at all. Considering the longer duration of surgery and the higher costs of the U-Blade, this result does not justify U-blade (RC) lag screw use^7^. The cut-out rate and sliding distance of the Gamma3 U-blade lag screw were not superior to those associated with other lag screws in other studies, and the study was not detailed according to the classification of fractures^8,9^. According to these clinical studies, the best intramedullary nail for femur trochanteric region fractures remains controversial.

The aim of this study was to evaluate whether additional use of the U-blade is associated with a reduced cut-out rate and lower limb length inequality as well as the sliding distance in patients with AO/OTA31 A1.1–3.3 intertrochanteric fractures. The study hypothesis was that the U-Blade (RC) lag screw would reduce cut-out rate and sliding distance of the lag screw.

## Methods

This study has been reviewed and approved by the Institutional Review Board (IRB) of The Catholic University of Korea, St. Vincent’s Hospital. The institutional review board waived the informed consent for this study owing to its retrospective nature. All methods were carried out in accordance with the relevant guidelines and regulations.

This is a retrospective review of two case series involving different lag screws for fixation of intertrochanteric fractures. A comparative analysis of patients treated with a Gamma3 nail with or without U-Blade (RC) lag screw between January 2014 and December 2018 was performed. Propensity score matching (PSM) analysis was performed to adjust the confounding factors to reach similar baseline characteristics. Data were retrieved from our department’s database and completed by chart reviews.

A revision of the AO/OTA Fracture and Dislocation Classification was published in the January 2018 issue of the Journal of Orthopaedic Trauma^10^. This module and new classification were used.

Inclusion criteria were: (1) All ages; (2) AO/OTA31 A1.1-A3.3 femur fractures treated with Gamma3 Nail (Stryker Trauma GmbH, Schoenkirchen, Germany) and with either standard lag screws or U-Blade (RC) Lag Screw (Stryker Trauma GmbH, Schoenkirchen, Germany) from January 2014-December 2018; and (3) Radiologic and clinical data were available preoperatively, immediate postoperatively, and at one, three, six months and one year after surgery. All cases were defined as AO/OTA31 A1.1-A3.3 fractures and were also classified simultaneously by two observers, clinical fellows in hip and pelvis division; agreement was reached by consensus.

Exclusion criteria were: (1) AO/OTA31 A1.1 fracture without surgery; (2) Postoperatively non-ambulatory patients; (3) Patients with pathological fractures; (4) Patients treated with other implant or a long Gamma Nail (Stryker Trauma Korea); (5) Follow up loss including expired or no appropriate radiologic follow up; and (6) Patients needed additional open fixation or cerclage wires. This study aims to evaluate the effect of different lag screw types, in case of A1.1 fracture, if the fracture line extended over the lateral 2/3 and/or to the medial cortex in the coronal plane according to MRI findings, surgery was performed. Patients without surgery were excluded^11^.

Between January 2014 and December 2018, a total of 978 patients were admitted to our hospital due to the trochanteric region fracture. Though the optimal suitability of specific implants has been extensively debated, among the short cephalomedullary nails, gamma nail was the most used, and PFNA was the next favorite in this institution.

**Figure 2:**
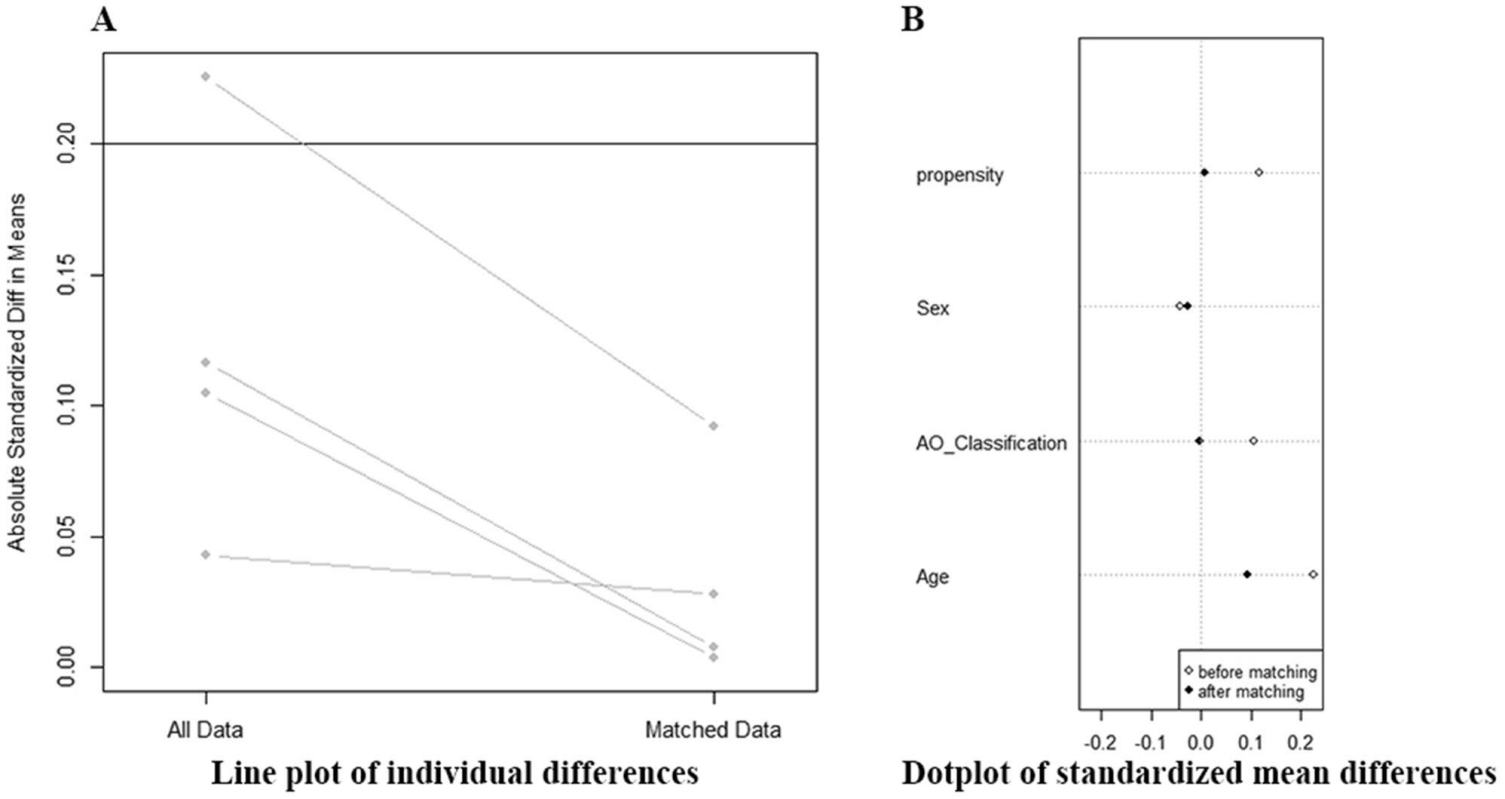
Evaluation of outcome of propensity score matching (PSM).

Among them, 531 cases were excluded (12 cases were classified as A1.1 fracture without surgery, 38 were non-ambulatory, 57 had pathologic fractures, long or other implants were used in 169 cases, 166 cases were excluded due to the follow up loss, and additional open fixation or cerclage wires were used in 89 cases). Of the 447 remaining, 152 patients received an additional anti-rotation U-Blade. Potential covariables included in PSM were age, gender and fracture classification. A total of 152 patients with U-Blade (group II) were matched to the patients with standard lag screw (group I). Line plot of individual differences and dotplot of standardized mean differences were shown to examine the outcome of PSM (Fig. 2). Finally, 304 patients in both groups were analyzed retrospectively. Patients’ demographics and preoperative diagnoses were collected as part of the history and physical examination, which was performed at the time of admission (Table 1).

**Table 1:** Patient demographics.

	Standard lag screw	U-blade lag screw	*p* value
Patients (n)	152	152	
Male/female	49/103	50/102	n.s. (0.903)
Age (mean, SD)	80.7 ± 7.6	81.4 ± 7.0	n.s. (0.443)
Side (R/L)	77/75	69/83	n.s. (0.358)
BMI (mean, SD)	22.7 ± 3.2	22.4 ± 3.4	n.s. (0.381)
NSA (mean, SD)	130.3 ± 7.5	129.1 ± 5.3	n.s. (0.115)
BMD (T-score)	− 2.90 ± 0.86	− 2.86 ± 0.93	n.s. (0.754)
ASA class (mean, median)	2.6, 3	2.5, 3	n.s. (0.083)
Preoperative Koval’s Grade (mean, SD)	2.7 ± 1.0	2.9 ± 1.1	n.s. (0.081)
Follow up (month) (mean, SD)	17.1 ± 7.6	14.2 ± 4.1	< 0.05 (0.000)
AO classification	152	152	
A1.1	2	2	
A1.2	26	27	
A1.3	38	37	
A2.2	32	32	
A2.3	34	34	
A3.1	4	5	
A3.2	7	6	
A3.3	9	9	n.s. (1.000)

### Operative technique

All operations were performed by the same surgeon, who had more than 20 years of experience in orthopaedic trauma. All patients were diagnosed by preoperative standard radiographs. Patients were positioned supine on the fracture table: the uninjured leg was placed on a leg holder. All patients received a standard 170 mm Gamma3 nail with standard lag screw or additional anti-rotation U-Blade of the appropriate length. A 125° lag screw angle was routinely used. The target lag screw placement was set in the middle/middle or inferior/middle position within the femoral head and the TAD or CalTAD was set to less than 25 mm to minimize the risk of cut-out^12,13^. Reaming of the medullary canal was generally performed in all cases before nail insertion. Tightening of the set-screw during the procedure was also done routinely and backed off a quarter turn to allow lag screw sliding. The use of an additional anti-rotation U-Blade was routinely done intraoperatively in group II. A distal locking screw was used.

In group I, a Gamma3 nail with standard lag screw was inserted. In group II of this study, a Gamma3 nail with lag screws with additional U-Blade (RC) was inserted. Implantation of the Gamma3 nail was performed under C-arm image intensifier in two planes. Additional open reduction or fixation with cerclage wires was not used.

The rehabilitation protocol consisted of one days of bed rest followed by ambulation with immediate weight bearing, eventually allowing for screw sliding and bone impaction. No other physiotherapy was suggested to the patients.

### Radiologic parameters

Since the hip joint geometry may not be influenced by side, the neck-shaft angle (NSA) was measured at the contralateral side before the operation^14^. Bone mineral density (BMD) was measured with dual energy X-ray absorptiometry (DEXA) in the trochanteric area on the contralateral side and reported as T-score. BMD measurements were possible in 136 patients, excluding 15 patients who had surgery on the contralateral side and one patient who did not have adequate BMD. In group II, measurements were possible in 138 patients, excluding 12 patients who had surgery on the contralateral side and two patients who did not have adequate BMD. For the same reason, NSA was measured in 137 patients in group I and 140 in group II.

On immediate postoperative X-ray, the accuracy of fracture reduction was measured according to Baumgaertner method^15^. Displacement of any fragment was more concentrated among the criteria. Radiographic observations assessed on postoperatively X-rays included visibility of fracture healing and bone healing, defined as the presence of a bridging callus and the absence of the fracture line. On the 12-months radiograph, lag screw sliding distance, reflecting limb length discrepancy, was assessed and compared between the two groups since the bone healing rate was reported as almost 100% by one year^16^.

**Figure 3:**
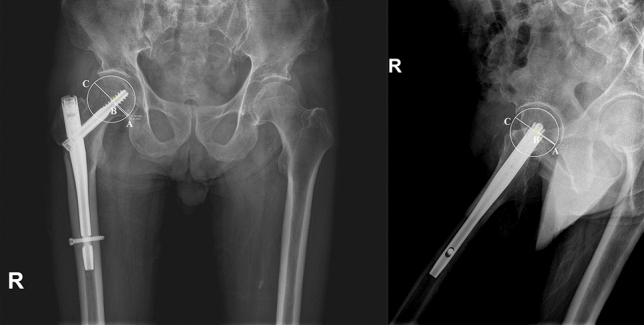
Assessment of the Parker’s ratio in the anterior–posterior plane and the lateral plane. The Parker’s ratio is represented by the percentage AB/AC** × **100.

**Figure 4:**
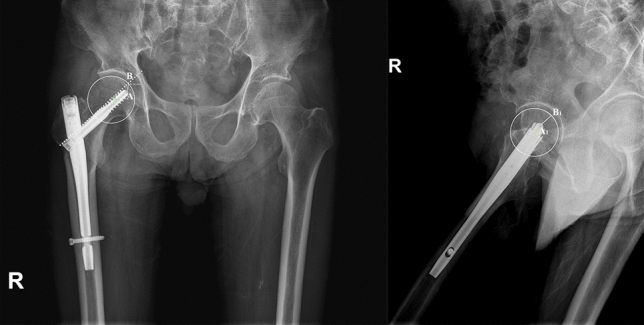
Assessment of the TAD in the anterior–posterior plane and the lateral plane. The Tip Apex Distance (TAD) is represented by the sum of AB and A_1_B_1_ distance.

**Figure 5:**
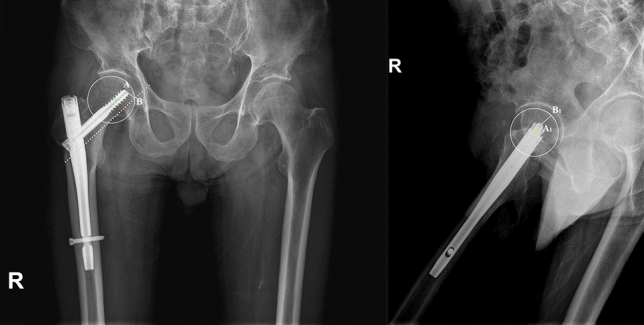
Assessment of the CalTAD in the anterior–posterior plane and the lateral plane. The Calcar Tip Apex Distance (CalTAD) is represented by the sum of AB and A_1_B_1_ distance.

**Figure 6:**
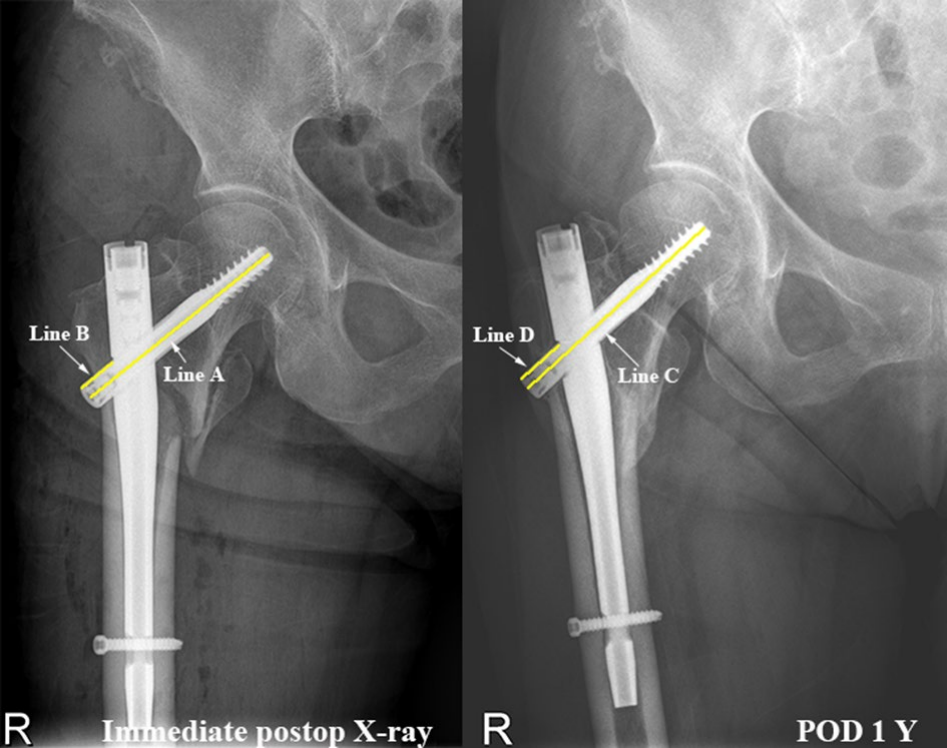
Actual amount of sliding in mms = Actual length of lag screws (D/C-B/A).

Parker’s ratio was used to categorize the placement of the lag screw in the femoral head in both the anterior–posterior plane and the lateral plane^11^ (Fig. 3). The likelihood of cutout was assessed by the tip-apex distance (TAD) (Fig. 4) and calcar referenced tip-apex distance (CalTAD) (Fig. 5) on postoperative radiographs^13,17^. Cut-out was defined as projection of the lag screw of more than 1 mm of the femoral head^11,15^. At the same time, we used a special radiographic method suggested by Lunsjo et al. and Serrano et al. to calculate the distance of lag screw sliding^18,19^. The sliding distance was calculated as the difference between the initial location and the location on the one-year X-ray (Fig. 6).

Follow ups were performed one, three, six months and one year postoperatively. At each postoperative visit, radiographs were made and any change in the position of the implant, complication, or fixation failure was recorded. All measurements were made twice a week by two observers and the means were finally recorded.

All implant related failures such as nail breakage, lateralization, migration, penetration, cut-out of lag screw, or other complications such as osteonecrosis, non-union, infection, and peri-implant fracture were reported during the follow up period. In addition, Harris hip score for pain and function and pre- and postoperative Koval’s grade at one year were evaluated.

### Statistical analysis

Pearson’s chi-square test was used to compare the differences in the categorical demographic variables between the two groups. The independent samples Student t-test or Mann–Whitney U test were used to compare continuous variables according to normality or sample size.

Sample size was estimated by utilizing an effect size of 0.5, an acceptable alpha error of 0.05, and a beta error of 0.2 to ensure power of 80%. Calculations indicated that it would be necessary to include at least 64 patients to compare means between two groups using independent samples Student’s t-test.

The Kolmogorov–Smirnov and Shapiro–Wilk tests were used to assess normality of distribution. Reproducibility was assessed based on the intraclass correlation coefficient (ICC). Intra-observer reliability was assessed using the values measured by each examiner. Inter-rater reliability was also measured by comparing the means of two observers. Reliability measurements were reviewed and the results reached substantial to almost perfect agreement (supplementary information).

Statistical analysis was performed using SPSS 25 software (SPSS, Inc., Chicago, IL, USA).

### Ethical approval

This research has been reviewed and approved by the Institutional Review Board (IRB) of the authors’ affiliated institutions (VC20RISI0038).

### Informed consent

Informed consent was waived for this study owing to its retrospective nature.

## Results

In both groups, patients experienced a mean delay of 3.1 days from initial admission to surgery and were operated on under general anesthesia. Mean follow-up was 15.6 ± 6.3 months, with a minimum of at least 12 months.

Basic patient demographics did not significantly differ between groups, with the exception of follow-up period. Follow-up period was significant different between the two groups (17.1 and 14.2 months, respectively; *p* < 0.05). Mean contralateral T-score measured using DEXA was not significantly different (*p* = 0.754). There was no significant difference in ambulatory ability before and after surgery as assessed with Koval’s grade (preoperative: *p* = 0.081, postoperative: *p* = 0.740). There was no significant distribution difference between the groups based on the new AO classification of fracture type (*p* = 1.000). More details are given in Tables 1 and 2.

**Table 2:** Comparison of patients between the standard and U-blade lag screw group.

	Standard	U-blade lag screw	*p* value
Surgical time (min)	54.1 ± 11.5	56.9 ± 14.2	n.s. (0.060)
Hospital stay (days)	15.4 ± 7.4	17.0 ± 9.9	n.s. (0.123)
Fracture reduction gap (mm)	2.9 ± 1.3	3.3 ± 2.3	n.s. (0.067)
Parker’s ratio AP 0	42.4 ± 6.3	43.7 ± 6.0	n.s. (0.057)
Parker’s ratio Lat 0	46.5 ± 6.1	45.7 ± 5.3	n.s. (0.207)
Parker’s ratio cut AP 0	43.4 ± 13.7	–	
Parker’s ratio cut Lat 0	44.2 ± 7.9	–	
TAD 0	19.5 ± 4.7	20.3 ± 5.2	n.s. (0.119)
TAD cut 0	20.2 ± 5.2	–	
CalTAD 0	20.6 ± 4.6	21.6 ± 4.9	n.s. (0.072)
CalTAD cut 0	20.9 ± 0.9	–	
Lateralization (12 month) (≥ 10 mm)	12 (7.9%)	5 (3.3%)	n.s. (0.081)
Sliding distance	5.6 ± 3.2 (n = 149)	4.6 ± 2.4 (n = 152)	< 0.05 (0.004)
A1	4.0 ± 2.4 (n = 65)	3.4 ± 2.2 (n = 66)	n.s. (0.124)
A2	6.1 ± 2.7 (n = 65)	5.2 ± 2.2 (n = 66)	< 0.05 (0.042)
A3	9.0 ± 4.3 (n = 19)	6.7 ± 1.5 (n = 20)	< 0.05 (0.034)
Cut-out rate	3 (2.0%)	0 (0.0%)	n.s. (0.082)
Time to cut-out (weeks) (mean, SD)	8.2 ± 1.5	–	–
Other complications	ONFH (1), Distal locking screw breakage (1)	Non-union (1), Distal locking screw breakage (1)	
Koval’s grade in 1 year (mean, SD)	3.4 ± 1.1	3.3 ± 1.3	n.s. (0.740)
HHS (1 year)	74.9 ± 8.6	76.7 ± 8.9	n.s. (0.076)

Since more time is required to place the additional U-blade during surgery, the mean duration of surgery was naturally slightly greater in group II, but the difference was not significant. Surgical time was 54.1 ± 11.5 min in group I and 56.9 ± 14.2 min in group II. There was also no difference in mean length of hospital stay in the two groups. The mean ASA score was higher in group I, but with no significant difference. Although the ASA score was high in group I, it was rather less likely to affect long-term hospitalization. Also, no significant difference was observed in functional outcome according to Harris hip score (HHS) at one year postoperative. More details are given in Table 2.

Most postoperative radiologic parameters were not significantly different between groups (Table 2). There was no significant difference in the two groups in the accuracy of reduction displacement by Baumgaertner classification. There were also no significant differences in Parker’s ratio of lag screw on the anteroposterior and lateral views on postoperative X-rays. In group I, there was no difference when comparing the Parker’s ratio in patients with versus without a cut-out of the lag screw (AP: *p* = 0.904, Lateral: *p* = 0.511). Similarly, there was no significant difference in TAD and CalTAD of lag screw between the groups. Also, no difference was observed when comparing TAD and CalTAD in patients with or without a cut-out of the lag screw in group I (TAD: *p* = 0.779, CalTAD: *p* = 0.904).

**Figure 7:**
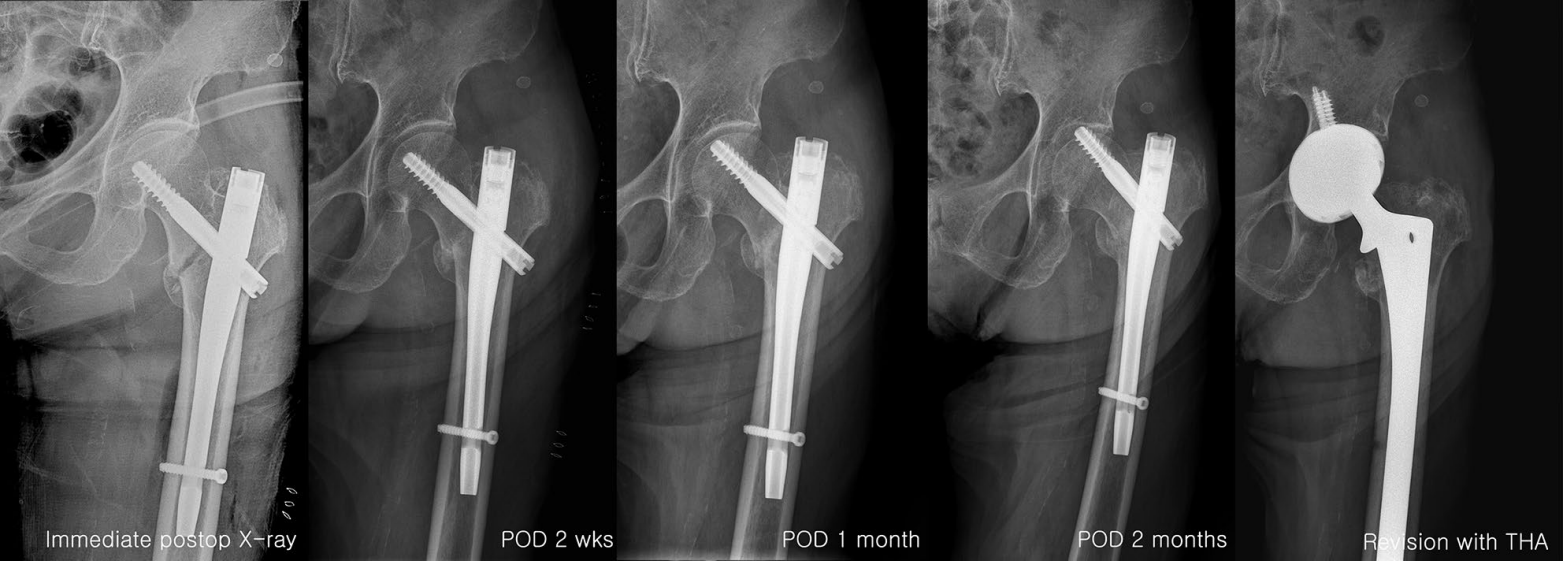
Cut-out example (Patient 2).

A total of three (2.0%) patients had a cut-out of the standard lag screw in group I. The cut-out was only observed in patients who treated standard lag screw and occurred 6–10 weeks after surgery. However, there was no significant difference between the two groups. These three patients experienced severe destruction of the acetabulum and underwent revision with total hip arthroplasty (Fig. 7).

**Figure 8:**
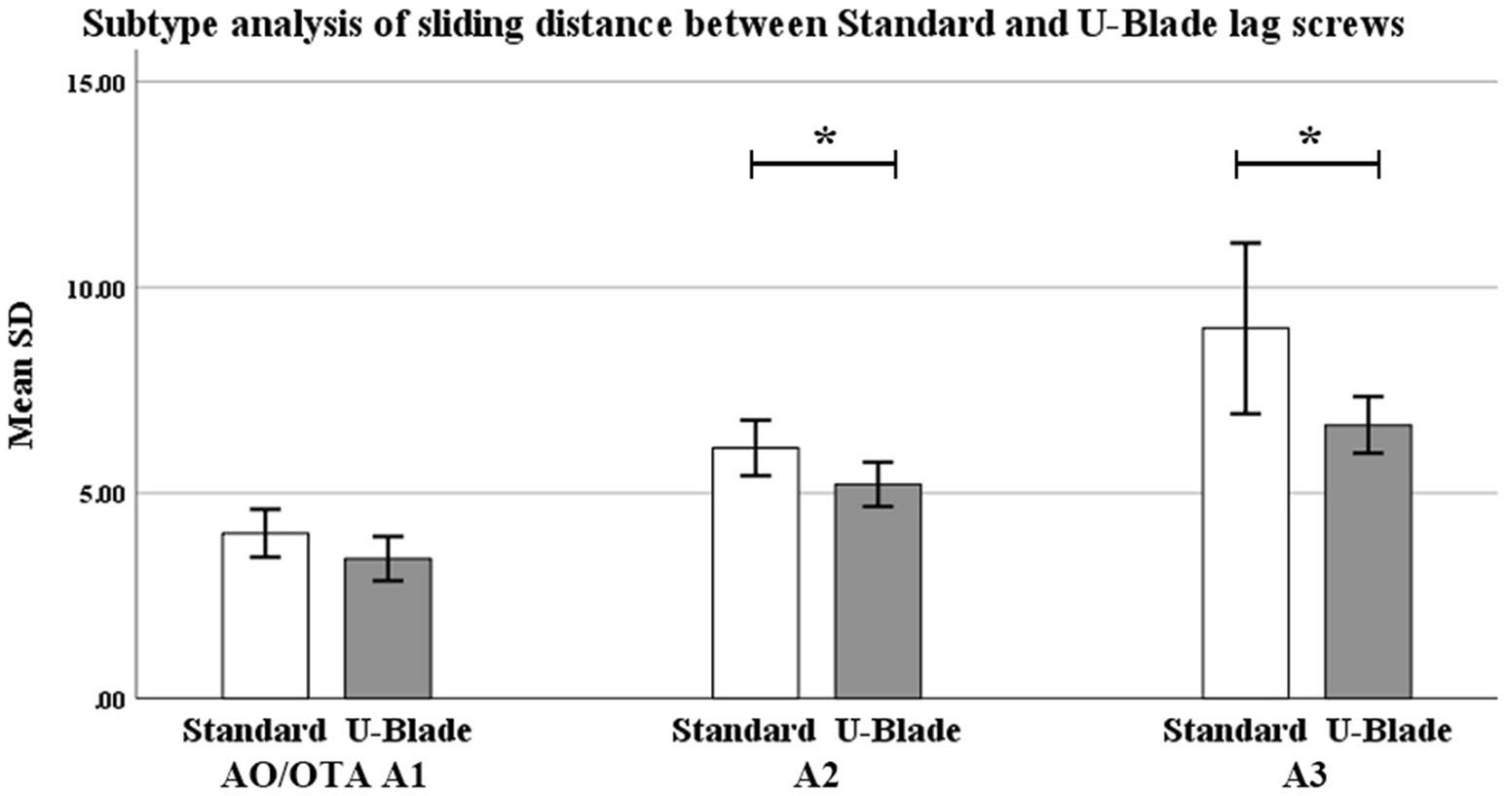
Subtype analysis of sliding distance between the groups in one year. * Significantly different in A2 and A3 at *p* < 0.05.

It is noteworthy that sliding distance and lag screw lateralization of more than 1 cm were significantly lesser prevalent in the U-blade lag screw group (5.6 ± 3.2 mm in group I, 4.6 ± 2.4 mm in group II; *p* < 0.05). In the subgroup analysis, this sliding distance was significantly longer in AO/OTA A2 and A3 fractures between the two groups. However, the difference in sliding distance lacks statistical significance in AO/OTA A1 fractures (Fig. 8). More details are given in Table 2.

A total of 17 patients showed a lateralization of the lag screw, 12 patients in group I and 5 in group II. Six of these 17 patients suffered from lag screw protrusion and soft tissue irritation due to lag screw lateralization, but these patients had a high prevalence of comorbidities and their symptoms were tolerable; none of these patients needed revision surgery or implant removal. There were four other complications: one ipsilateral femoral head osteonecrosis, two distal locking screw breakages, and a non-union of the fracture during the study period. There was one patient with ipsilateral osteonecrosis and two with distal locking screw breakage postoperatively, neither of whom required any further management. Non-union, a well-known but rare complication, occurred in one patient in the U-blade Gamma3 group. Revision bipolar hemiarthroplasty was performed after removal of the nail.

## Discussion

The additional use of U-blade seems to be associated with lower sliding distance but not lower cut-out rate.

The cut-out rate was known to be about 3.5% when surgeons used various lag screw implants. In the literature, a total of 141 events were reported from 3692 cases^20^. However, U-blade hybrid type lag screw failure has rarely been reported^21^. In the literature, the cut-out rates were reported to be about 0–3.3% with the use of an additional U-blade^7,9,22–24^. In this study, the cut-out rate was 2.0% with the use of Gamma3 nail standard lag and 0% with the additional use of a U-blade, both of which were within the expected range. However, follow up loss rate was 17.0%, so the actual cut-out rate may be somewhat higher.

Patients with hip fractures are usually recommended operative treatment. Surgical management enables earlier mobilization and results in better functional outcomes. However, even among patients who undergo operations, the one-year mortality rate is as high as 6.6–36.4%^11,25,26^. A certain level of follow-up loss is unavoidable in this situation, which causes statistical bias. In the propensity model, we tried to adjust the confounding factors and reduce the bias after reaching similar baseline characteristics.

The difference in sliding distance varied from study to study. Lateralization is inherent to a healing process and reflects the sliding distance, and a previous study that used an additional U-blade showed no significant difference between that and a standard lag screw^7^. In another study, the sliding distance associated with a Gamma3 nail lag screw with U-blade was found to be between that of other implant lag screws^9^. Other studies yielded favorable outcomes of a U-blade^22,24^. In the present study, the sliding distance was significantly shorter in the U-blade Gamma3 nail group than in the standard lag screw group, in A2 and A3 fractures. This result is consistent with the biomechanical study in which the U-blade lag screw resulted in increased resistance by enlarging the contact surface around the femoral head. These findings can help surgeons choose the proper implant to reduce leg length inequality.

U-blade lag screw resulted in increased resistance by enlarging the contact surface around the femoral head, neck and intertrochanteric region between screw and bone. It is predictable that the degree of sliding of the U-blade lag screw with high resistance is lower than that of the standard lag screw in intertrochanteric fractures. The difference in lag screw resistance may play a significant role when fractures coalesce. In the subgroup analysis, the sliding distance was significantly different in A2 and A3 fractures between the groups. In A2 and A3 fractures, sliding distance was significantly reduced when a U-blade was used. However, in A1 fractures, it could be considered that the lag screw difference did not play a big role because the sliding distance was not large than A2 and A3 fractures from the beginning.

The rate of lag screw sliding is known to be greater when the fracture pattern is unstable^27^. As expected in this study, in unstable intertrochanteric fractures such as AO/OTA A2, A3 fractures, the mean lag screw sliding distance was greater than that seen in A1 fractures in both groups.

In the 2018 AO/OTA fracture and dislocation classification, lateral wall height or thickness is defined as the distance in millimeters (mm) from a reference point 3 cm below the innominate tubercle of the greater trochanter angled 135° upward to the fracture line on the anteroposterior X-ray. In the literature, a reliable indicator of postoperative lateral wall fractures was lateral wall thickness and the threshold point was 20.5 mm^28,29^. A2 fractures were associated with a greater risk of postoperative wall fracture and reoperation due to fixation failure than A1 fractures. Sliding distance is most dependent on the fracture type and reduction quality and may have a fundamental correlation with the degree of collapse in fusion fractures. Thus, the increased sliding distance seen in A2 and A3 fractures as compared with A1 fractures may be a general result.

Furthermore, in addition to the unstable fracture pattern, it is known that the calcar gapping is associated with more sliding distance^27,30^. Since both study groups had similar fracture reduction displacement (n.s.), it was difficult to draw clear conclusions about this variable.

Lag screw sliding may help bone union by stimulating callus formation when initial weight bearing is permitted with increasing bone impaction and stability at the fracture site. However, lag screw sliding distance and collapse can affect lower limb length inequality, though of course sliding distance is not the only one factor which can do so. Sliding distance is also closely related to the limb length discrepancy. Cephalad migration and axial movement in the femoral head also need to be evaluated to calculate de novo limb length mismatch^11^. Since measuring all relevant parameters is hard, scanograms of both lower extremities are needed to properly evaluate limb length discrepancy, and this subject deserves further investigated.

Intertrochanteric fracture shortening and collapse were significantly associated with limb length inequality, persistent pain, and altered hip mechanism^31,32^. Thus, reducing the sliding distance has many advantages that should not be overlooked. In the literature, the excessive sliding of the lag screw of a short cephalomedullary nail does not improve any clinical results and certain cases, such as highly comminuted A1 and A2 fractures, can even benefit from a locked lag screw by tightening the set-screw^16^. Other lag screws with more resistance and shorter sliding distance such as dual screws can be considered to treat the proximal femur intertrochanteric fractures^9,19^.

Other surgical complications may also occur, such as femoral head osteonecrosis, non-union and distal locking screw breakage. In patients who are operated upon with closed reduction and internal fixation, femoral head osteonecrosis and non-union are known to be uncommon^33,34^. Also, since there is an ongoing debate as to whether a distal locking screw is necessary, we can predict that distal locking screw breakage during fracture healing dose not impair bone healing and will not cause other clinical complications^35^. Different decisions should be made in the management of complications according to each patient’s individual scenario.

There are several studies on the sliding distance of cephalomedullary nails, and none have included subtype analysis. However, several limitations of the present study should be considered. As mentioned above, in exclusion criteria, many patients were lost to follow-up. We had to exclude 17.0% of our geriatric patients because of the incomplete follow-up. In addition, different implants were used at a certain proportion. Even in the matching model, for these reasons, selection bias cannot be ruled out completely.

Second, although there were enough cases to evaluate differences using independent sample t-test, the sample size was relatively small, especially when attempting subtype analysis. The price of a U-blade is approximately 1.2 times higher than that of a standard lag screw in the authors’ country. In the other countries, the price may be more expensive. Some authors have reported the price of an additional U-blade as 2.5 times higher than that of a standard lag screw. Finally, in this situation, with uncertain results, there were ethical limits to conducting retrospective or prospective studies.

In conclusion, the U-Blade (RC) lag screw did not reduce the cut-out rate in treatment of trochanteric region fractures. However, there was a significant difference in lag screw sliding such that the U-Blade (RC) lag screw was associated with a shorter sliding distance and more resistance in AO/OTA31 A2 and A3 fractures. However, the difference in sliding distance lacks statistical significance in AO/OTA A1 fractures. These findings can help trauma surgeons choose the proper implant to reduce leg length inequality.

## Supplementary Information

Supplementary Information.
