# Supplementary material for: Additional use of anti-rotation U-blade (RC) decreases lag screw sliding and limb length inequality in the treatment of intertrochanteric fractures
Source: Sci Rep. 2021 Aug 31;11:17417. doi: 10.1038/s41598-021-96988-z (PMC8408211; doi:10.1038/s41598-021-96988-z)
Supplement: Supplementary file 1 — Supplementary Information. [file 41598_2021_96988_MOESM1_ESM.docx]

**Supplementary information**

The intraclass correlation coefficients (ICC) with 95% confidence interval (CI) and of intra-observer reliability

| Examiner 1 | ICC by examiner 1 | 95% CI |
| --- | --- | --- |
| Neck-Shaft Angle (NSA) | 0.94 | -0.05-0.99 |
| Fracture reduction displacement | 0.93 | 0.00-0.98 |
| Parker’s ratio AP 0 | 0.82 | -0.02-0.96 |
| Parker’s ratio Lat 0 | 0.69 | 0.50-0.80 |
| Parker’s ratio cut AP 0 | 0.99 | 0.09-1.00 |
| Parker’s ratio cut Lat 0 | 0.97 | 0.16-1.00 |
| TAD 0 | 0.74 | -0.01-0.90 |
| TAD cut 0 | 0.97 | 0.02-1.00 |
| CalTAD 0 | 0.85 | 0.27-0.94 |
| CalTAD cut 0 | 0.98 | 0.34-1.00 |
| Sliding distance | 0.82 | 0.78-0.86 |
| Examiner 2 | ICC by examiner 2 | 95% CI |
| Neck-Shaft Angle (NSA) | 0.91 | -0.05-0.98 |
| Fracture reduction displacement | 0.97 | 0.88-0.99 |
| Parker’s ratio AP 0 | 0.71 | 0.49-0.82 |
| Parker’s ratio Lat 0 | 0.61 | 0.31-0.76 |
| Parker’s ratio cut AP 0 | 0.99 | 0.36-1.00 |
| Parker’s ratio cut Lat 0 | 0.97 | 0.48-1.00 |
| TAD 0 | 0.76 | -0.05-0.91 |
| TAD cut 0 | 0.93 | 0.01-1.00 |
| CalTAD 0 | 0.83 | 0.75-0.87 |
| CalTAD cut 0 | 0.82 | -0.04-0.99 |
| Sliding distance | 0.73 | 0.66-0.78 |

The intraclass correlation coefficients (ICC) with 95% confidence interval (CI) of inter-observer reliability

| Examiner 1 and 2 | ICC | 95% CI |
| --- | --- | --- |
| Neck-Shaft Angle (NSA) | 0.89 | -0.11-0.97 |
| Fracture reduction displacement | 0.98 | 0.87-0.99 |
| Parker’s ratio AP 0 | 0.94 | 0.92-0.95 |
| Parker’s ratio Lat 0 | 0.85 | 0.82-0.88 |
| Parker’s ratio cut AP 0 | 0.99 | 0.84-1.00 |
| Parker’s ratio cut Lat 0 | 0.98 | 0.67-1.00 |
| TAD 0 | 0.96 | 0.95-0.97 |
| TAD cut 0 | 0.99 | 0.39-1.00 |
| CalTAD 0 | 0.93 | 0.87-0.96 |
| CalTAD cut 0 | 0.91 | 0.00-1.00 |
| Sliding distance | 0.89 | 0.86-0.91 |

Interpretation of kappa and intraclass correlation coefficient according to Fleiss and Viera and Garrett ^1^

| Kappa Statistic | Strength of Agreement beyond Chance |
| --- | --- |
| < 0 | Poor agreement |
| 0-0.20 | Slight agreement |
| 0.21-0.40 | Fair agreement |
| 0.41-0.60 | Moderate agreement |
| 0.61-0.80 | Substantial agreement |
| 0.81-1.00 | Almost perfect agreement |

1 Landis, J. R. & Koch, G. G. The measurement of observer agreement for categorical data. *Biometrics* **33**, 159-174 (1977).
